# Computational Recognition and Clinical Verification of TGF-β-Derived miRNA Signature With Potential Implications in Prognosis and Immunotherapy of Intrahepatic Cholangiocarcinoma

**DOI:** 10.3389/fonc.2021.757919

**Published:** 2021-10-25

**Authors:** Zaoqu Liu, Siyuan Weng, Hui Xu, Libo Wang, Long Liu, Yuyuan Zhang, ChunGuang Guo, Qin Dang, Zhe Xing, Taoyuan Lu, Xinwei Han

**Affiliations:** ^1^ Department of Interventional Radiology, The First Affiliated Hospital of Zhengzhou University, Zhengzhou, China; ^2^ Interventional Institute of Zhengzhou University, Zhengzhou, China; ^3^ Interventional Treatment and Clinical Research Center of Henan Province, Zhengzhou, China; ^4^ Department of Hepatobiliary and Pancreatic Surgery, The First Affiliated Hospital of Zhengzhou University, Zhengzhou, China; ^5^ Department of Endovascular Surgery, The First Affiliated Hospital of Zhengzhou University, Zhengzhou, China; ^6^ Department of Colorectal Surgery, The First Affiliated Hospital of Zhengzhou University, Zhengzhou, China; ^7^ Department of Neurosurgery, The Fifth Affiliated Hospital of Zhengzhou University, Zhengzhou, China; ^8^ Department of Cerebrovascular Disease, Zhengzhou University People’s Hospital, Zhengzhou, China

**Keywords:** intrahepatic cholangiocarcinoma, microRNAs, TGF-β, prognosis, recurrence, immunotherapy

## Abstract

MicroRNAs (miRNAs) were recently implicated in modifying the transforming growth factor β (TGF-β) signaling in multiple cancers. However, TGF-β-derived miRNAs and their potential clinical significance remain largely unexplored in intrahepatic cholangiocarcinoma (ICC). In this study, we proposed an integrated framework that enables the identification of TGF-β-derived miRNAs in ICC (termed “TGFmitor”). A total of 36 TGF-β-derived miRNAs were identified, of which nine significantly correlated with overall survival (OS) and aberrantly expressed in ICC. According to these miRNAs, we discovered and validated a TGF-β associated miRNA signature (TAMIS) in GSE53870 (n =63) and TCGA-CHOL (n =32). To further confirm the clinical interpretation of TAMIS, another validation based on qRT-PCR results from 181 ICC tissues was performed. TAMIS was proven to be an independent risk indicator for both OS and relapse-free survival (RFS). TAMIS also displayed robust performance in three cohorts, with satisfactory AUCs and C-index. Besides, patients with low TAMIS were characterized by superior levels of CD8+ T cells infiltration and PD-L1 expression, while patients with high TAMIS possessed enhanced CMTM6 expression. Kaplan-Meier analysis suggested CMTM6 could further stratify TAMIS. The TAMIS^high^CMTM6^high^ subtype had the worst prognosis and lowest levels of CD8A and PD-L1 expression relative to the other subtypes, indicating this subtype might behave as “super-cold” tumors. Notably, the improved discrimination was observed when CMTM6 was combined with TAMIS. Overall, our signature could serve as a powerful tool to help improve prognostic management and immunotherapies of ICC patients.

## Introduction

Intrahepatic cholangiocarcinoma (ICC) is a primary malignant tumor with high heterogeneity and invasiveness, mainly originating from the secondary bile duct (1). Over the past few decades, the incidence of ICC has increased by up to 10-fold globally (1–3). Given the unfavorable prognosis of ICC, mortality should parallel incidence rates (1). Surgical resection remains the mainstay of latently curative treatment for ICC, with median relapse-free survival (RFS) durations of 1-3 years (4, 5). Recently, immunotherapies that function by targeting immune checkpoints have achieved encouraging progression in cancer treatment (6). However, to date, only a subset of patients yields considerable benefit in ICC (7). Therefore, early intervention for the “high-risk” ICC is vital to improve clinical outcomes of patients and searching for new ways to stratify ICC patients is imperative.

Transforming growth factor β (TGF-β) signaling is an important intracellular signal transduction pathway, playing crucial roles in tumor initiation and progression (8, 9). Previous study has demonstrated that TGF-β signaling is closely involved in the invasion, metastasis, and recurrence (10). Moeini A et al. have reported that compared with the stem-cell and classical types of hepatobiliary carcinoma, ICC is characterized by chromosomal stability and active TGF-β signaling (11). Besides, accumulating evidence has revealed that TGF-β is a crucial enforcer of immune homeostasis and tolerance, exerting systemic immune suppression and inhibiting host immune surveillance (8, 9). For instance, inhibition of IL10 and TGF-β receptors on dendritic cells could facilitate the activation of effector T cells to eliminate cholangiocarcinoma cells (12), highlighting the essential roles of TGF-β in ICC.

Small noncoding microRNAs (miRNAs) are endogenous molecules that participate in the post-transcriptional modification process (13). Previous studies have established close connections between aberrant miRNA expression and TGF-β signaling in multiple cancers (14–16). However, TGF-β-derived miRNAs and their potential clinical significance remain largely unexplored in ICC.

In this study, we proposed an integrated framework that enables the identification of TGF-β-derived miRNAs (termed “TGFmitor”). A TGF-β associated miRNA signature (TAMIS) was constructed and validated in two independent datasets and a clinical in-house cohort. The immune landscape and immune checkpoint profiles of TAMIS were also investigated. Initial establishment and immune characterization of TAMIS for stratifying risk might help improve prognostic management and immunotherapies of ICC patients.

## Materials and Methods

### Publicly Available Data Collection and Processing

**Figure 1:**
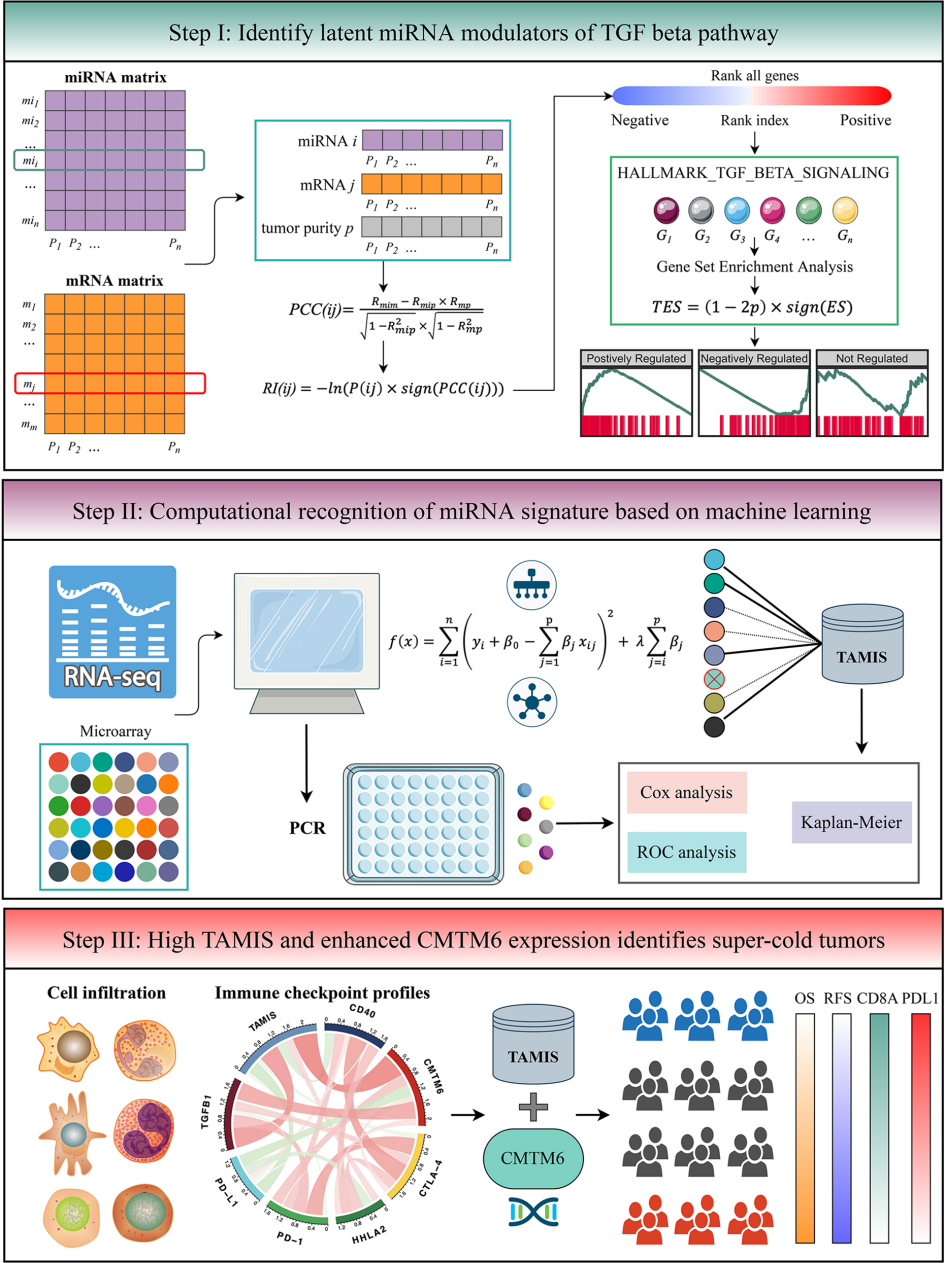
The flowchart of this study.

The flowchart of this study is shown in **Figure 1**. Two independent cohorts were enrolled from The Cancer Genome Atlas (TCGA), and Gene Expression Omnibus (GEO) datasets, including TCGA-CHOL and GSE53870. Patients were retained if they: (1) were primary ICC; (2) possessed both miRNA expression profiles and prognostic information (mRNA expression data was also required in TCGA-CHOL); (3) no preoperative chemotherapy or radiotherapy received. The detailed baseline data was summarized in **Table S1**. For TCGA-CHOL, the mRNA and miRNA raw read count from the TCGA portal was transformed into transcripts per kilobase million (TPM) and reads per kilobase million (RPM), respectively. The GENCODE (https://www.gencodegenes.org/) and miRBase (http://www.mirbase.org/) were applied to mRNA and miRNA annotations, respectively. For GSE53870, we directly downloaded the series matrix files and further performed quantile normalization. The GPL18118 (State Key Laboratory Human microRNA array 1104) was utilized to miRNA annotation. The miRNAs with zero expression value >50% of the samples in each dataset were excluded. Further, the miRNA intersection of two datasets was taken, and ultimately, a total of 560 miRNAs were retained for the subsequent analysis.

### Hallmark Gene Sets

Fifty Hallmark gene sets were retrieved from the MSigDB resource (version 7.4, h.all.v7.4.symbols.gmt), which summarize and represent specific well-defined biological states or processes and demonstrate coherent expression (17). These gene sets were extensively utilized in cancer-related studies (18–22).

### Gene Set Variation Analysis (GSVA)

To reveal the difference on Hallmark biological process between tumor and normal samples, we performed GSVA enrichment analysis *via* “GSVA” R package. GSVA is an unsupervised and non-parametric algorithm that quantifies the activity variation of each pathway over a sample population (23). The “limma” R package was employed to identify significant pathways with false discovery rate (FDR) <0.05 and absolute value of log2 (fold change) >0.5. Univariate Cox analysis further revealed the prognostic significance of these significant pathways, and pathways with *P <*0.05 were used for the next exploration.

### TGFmitor: Identifying the Potential miRNA Modulators of TGF-β Signaling

To identify the potential miRNA modulators of TGF-β signaling, we developed an integrated pipeline with reference to previous studies (13, 24). Briefly, all mRNAs were sorted in descending order *via* their correlation with a specific miRNA (adjusted by tumor purity). The ordered gene list was further subjected to “fgsea” R package to explore whether the genes of TGF-β signaling were enriched in the top or bottom of the ordered list. The TGF-β enrichment score (*TES*) was measured for all miRNAs. According to the permutation test framework, miRNAs with significant TES were determined as the TGF-β-derived miRNAs.

As displayed in the step I of flowchart (**Figure 1**), for miRNA and mRNA expression matrixes, miRNA *i* and mRNA *j* within *n* patents were defined as *MI(i)* = (*mi_1_*, *mi_2_*, …, *mi_n_*) and *M(j)* = (*m_1_*, *m_2_*, …, *m_n_*), respectively. The “ESTIMATE” R package was applied to quantify tumor purity across *n* patients, which were defined as *P* = (*p_1_, p_2_, …, p_n_*). The first-order partial correlation coefficient (PCC) between miRNA *i* and mRNA *j* was calculated by removing the effects of tumor purity:

$PCC\left(ij\right)=\frac{R_{mim}-R_{mip\;}\times \;R_{mp}}{\sqrt{1-R_{mip}^{2}}\;\times \;\sqrt{1-R_{mp}^{2}}}$

*R_mim_*, *R_mip_*, and *R_mp_* were defined as the Pearson correlation coefficients between miRNA *i* and mRNA *j*, miRNA i and tumor purity *p*, as well as mRNA *j* and tumor purity *p*, respectively. Next, the *P*-value of *PCC(ij)* labeled as *P(ij)* was measured as followed:

$P\left(ij\right)=2\times \;pnorm\left(-\left|PCC_{ij}\times \;\sqrt{\frac{n-3}{1-\;PCC_{ij}^{2}}}\right|\right)$

*pnorm* was the normal distribution function and *n* was defined as the number of samples. For miRNA *i*, the rank index (*RI*) of mRNA *j* was calculated:

RI(ij)= −ln(P(ij) × sign(PCC(ij)))

*sign* function was an odd mathematical function that extracts the signs of *PCC(ij)*. Based on the descending order of *RI*, all mRNAs were ranked and further subjected to gene set enrichment analysis (GSEA). The genes of TGF-β signaling were mapped to the ordered gene list. For miRNA *i*, the enrichment score (*ES*) and *P*-value (adjusted by FDR) were estimated *via* “fgsea” R package, and then merged into a *TES*:

$TES\left(i\right)=\left(1-2P_{i}\right)\times \;sign\left(ES_{i}\right)$

Thus, *TES* ranged from -1 to 1. Following previous studies (13, 24), the miRNAs with the absolute value of *TES >*0.995 and FDR <0.05 were determined as TGF-β-derived miRNAs.

### Signature Generation

Prior to generate a TGF-β associated miRNA signature (TAMIS), we transformed miRNA expression into z-score in both TCGA-CHOL and GSE53870 cohorts, which enhanced the comparability between different datasets. Based on the expression profiles of TGF-β-derived miRNAs, we performed univariate Cox analysis in two cohorts. Given the strictness of multiple testing correction and the small size samples might filter out some potential miRNAs associated with overall survival (OS), we selected miRNAs with both unadjusted *P*-value <0.1 and the same hazard ratio (HR) direction for constructing the TAMIS model *via* LASSO algorithm. The initial signature discovery was performed in GSE53870 and then independently validated in TCGA-CHOL. Using the leave-one-out cross-validation (LOOCV) framework, the optimal lambda in LASSO was generated when the partial likelihood deviance reached the minimum value. The key miRNAs with nonzero coefficients were included to fit a predictive signature. The TAMIS formula was calculated with the LASSO model weighting coefficient as follows:

$TAMIS\;score=\;\overset{n}{\underset{i=1}{\sum }}Expression_{i}\;\times \;Coefficient_{i}$

*n* was the number of key miRNAs, *Expression_i_* was the expression of miRNA* i*, and *Coefficient_i_* was the corresponding LASSO coefficient.

### Human Tissue Specimens

This project was approved by the Ethics Committee Board of The First Affiliated Hospital of Zhengzhou University. A total of 181 paired ICC tissues and their matched adjacent normal tissues were collected from The First Affiliated Hospital of Zhengzhou University. All patients gave written informed consent, and none of the patients received any preoperative chemotherapy or radiotherapy. The detailed baseline information of patients was displayed in **Table S1**.

### Quantitative Real-Time PCR (qRT-PCR)

Total RNA was isolated with RNAiso Plus reagent (Takara, Dalian, China) as described previously (19). RNA quality was evaluated using a NanoDrop One C (Waltham, MA, USA), and RNA integrity was assessed using agarose gel electrophoresis. An aliquot of 1 µg of total RNA was reverse-transcribed into complementary DNA (cDNA) using a High-capacity cDNA Reverse Transcription kit (TaKaRa Bio, Japan), according to the manufacturer’s protocol. miRNAs were reverse transcribed using a miRNA reverse transcription kit (TaKaRa Bio, Japan). The primer sequences were shown in **Table S2**. See Supplementary Material for details.

### Immunohistochemistry (IHC)

For IHC assay, paraffin sections were incubated with primary antibodies against CD8A (1:300; Servicebio, *catlog#GB11068-1*), PD-L1 (1:500, Servicebio, *catlog#GB11339A*) and CMTM6 (1:500, Abcam, *catlog#ab264067*) at 37°C for 60 mins, secondary antibodies at 37°C for 15 mins and horseradish enzyme labelled streptavidin solution for 10 mins, then stained by DAB and hematoxylin. Staining percentage scores were classified as follows: 1 (1%–25%), 2 (26%–50%), 3 (51%–75%) and 4 (76%–100%), and staining intensity was scored 0 (signalless color) to 3 (light yellow, brown, and dark brown). The stained tissues were scored by three individuals blinded to the clinical parameters, and the IHC scores were determined by percentage and intensity scores.

### Cells Infiltration

The single sample gene set enrichment analysis (ssGSEA) implemented in “GSVA” R package was employed to quantify the relative infiltration of 28 immune cells in ICC (25).

### Unsupervised Subclass Mapping (SubMap)

SubMap is an unsupervised method that employs GSEA approach to reveal common subtypes between independent datasets (26). In this study, the SubMap was utilized to evaluate the expression similarity between the two risk groups and the patients with a different immunotherapeutic efficacy. A Bonferroni adjusted *P <*0.05 suggests the significant similarity between two groups.

### TIDE and TIS Estimation

To assess the putative response to immune checkpoint inhibitors (ICIs) for each sample, we performed two algorithms, including Tumor Immune Dysfunction and Exclusion (TIDE) and T-cell inflammatory signature (TIS). TIDE (http://tide.dfci.harvard.edu/) combines two primary mechanisms of immune evasions: T cell dysfunction and T cell exclusion, to quantify tumor immune evasion. A higher TIDE score indicates the stronger potential of tumor immune evasion and worse immunotherapeutic efficacy (27). TIS is composed of 18 inflammatory genes associated with antigen presentation, chemokine expression, cytotoxic activity, and adaptive immune resistance (28). This signature was used to predict clinical response to PD-1 blockade.

### Statistical Analysis

All data processing, statistical analysis, and plotting were conducted in R 4.0.5 software.

The LASSO algorithm was fitted by “glmnet” R package. The “survminer” R package was used to determine the optimal cutoff value. Cox regression and Kaplan-Meier analysis were performed *via* “survival” R package. The time-dependent area under the ROC (AUC) for survival variable was conducted by “timeROC” R package. The calibration curve was plotted *via* “rms” R package. The “ggDCA” R package was utilized to perform the decision curve analysis (DCA). All statistical tests were two-sided. *P <*0.05 was regarded as statistically significant.

## Results

### TGF-β Signaling Was Upregulated and Correlated With Prognosis in ICC

**Figure 2:**
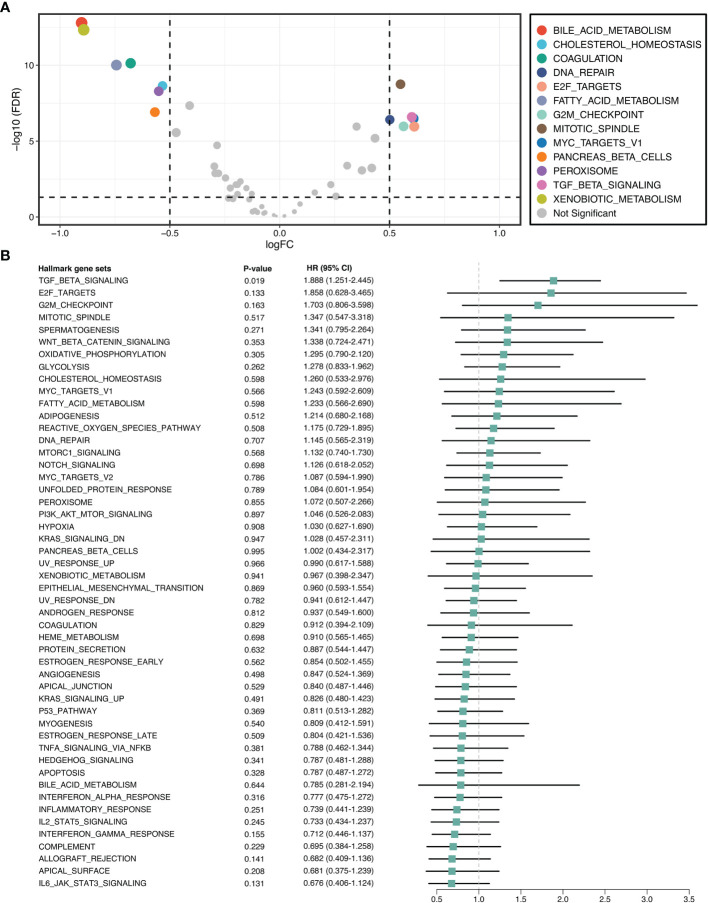
TGF-β signaling was upregulated and correlated with prognosis in ICC. **(A)** Differential analysis of the activity variation of fifty Hallmark gene sets between tumor and normal samples. **(B)** Univariate Cox regression analysis of fifty Hallmark gene sets in ICC.

GSVA quantified the activity variation of fifty Hallmark gene sets between tumor and normal samples. As displayed in **Figure 2A**, six pathways were significantly upregulated in ICC, namely DNA repair, E2F targets, G2M checkpoint, mitotic spindle, MYC targets V1, and TGF-β signaling; while seven pathways were dramatically downregulated, namely bile acid metabolism, cholesterol homeostasis, coagulation, fatty acid metabolism, pancreas beta cells, peroxisome, and xenobiotic metabolism. This suggested that cholesterol and phospholipid metabolisms were weakened, while proliferation and TGF-β were enhanced in ICC. Further univariate Cox regression revealed the prognostic value of these pathways, and notably, only TGF-β signaling was significant (HR =1.888 [1.251-2.455], *P* =0.019) (**Figure 2B**). Hence, we used TGF-β signaling for the next exploration.

### Identifying the Potential miRNA Modulators of TGF-β Signaling in ICC

**Figure 3:**
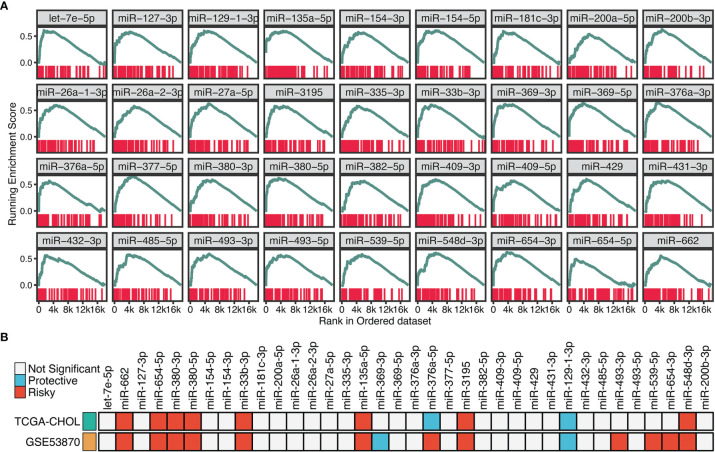
Identifying the potential miRNA modulators of TGF-β signaling in ICC. **(A)** The enrichment score (ES) distribution for 36 TGF-β-derived miRNAs. Each vertical bar represents a gene involved in the TGF-β signaling. **(B)** Univariate Cox regression analysis of 36 TGF-β-derived miRNAs in GSE53870 and TCGA-CHOL.

To identify the potential miRNA modulators of TGF-β signaling, we introduced a three-step framework termed “TGFmitor”. Based on the expression profiles of miRNA and mRNA, TGFmitor could systematically decipher candidate miRNA modulators of TGF-β signaling. A hypothesis is that if a specific miRNA is closely implicated in TGF-β signaling, genes within this pathway should be enriched in the top or bottom of the ordered gene list. In total, TGFmitor identified 36 TGF-β-derived miRNAs, which accounted for 6.4% of all miRNAs in two cohorts (**Figure 3A**). These miRNA modulators could serve as a resource for deciphering the TGF-β regulation in ICC. We further explored the concordance of these miRNAs in two cohorts and found nine miRNAs that stably associated with OS (**Figure 3B**). Subsequently, qRT-PCR analysis between 181 paired ICC tissues and their matched adjacent normal tissues further demonstrated the expression trends of these miRNAs, eight miRNAs including miR-135a-5p, miR-3195, miR-33b-3p, miR-380-3p, miR-380-5p, miR-548-3p, miR-654-5p, and miR-662 were significantly overexpressed, while only miR-129-1-3p was pronouncedly downregulated in ICC (**Figure S1**). Thus, these nine miRNAs demonstrated significant prognostic value and stable differential expression in ICC.

### Signature Generation Based on LASSO Algorithm and LOOCV Framework

**Figure 4:**
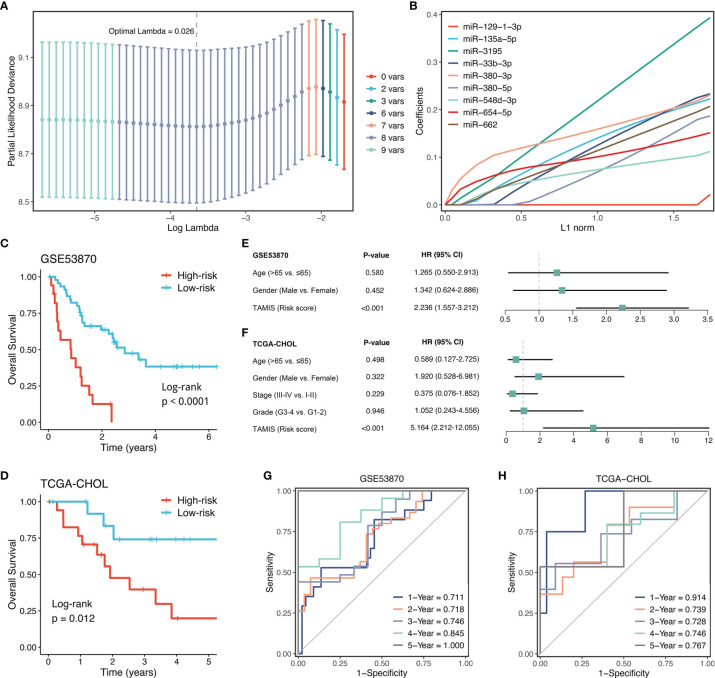
Construction and validation of TAMIS. **(A)** Determination of the optimal lambda was obtained when the partial likelihood deviance reached the minimum value. and further generated the key miRNAs with nonzero coefficients. **(B)** LASSO coefficient profiles of the candidate miRNAs for TAMIS construction. **(C, D)** Kaplan-Meier curves of OS according to TAMIS in GSE53870 **(C)** and TCGA-CHOL **(D–F)**. Multivariable Cox regression analysis of TAMIS in GSE53870 **(E)** and TCGA-CHOL **(F)**. **(G, H)** Time-dependent ROC analysis for predicting OS at 1, 2, 3, 4, and 5 years in GSE53870 **(G)** and TCGA-CHOL **(H)**.

With the expression profiles of these nine miRNAs, we fitted a LASSO model with LOOCV framework. According to the optimal lambda value (0.026), eight key miRNAs with nonzero coefficients were considered to be strongly predictive of OS, including miR-135a-5p, miR-3195, miR-33b-3p, miR-380-3p, miR-380-5p, miR-548-3p, miR-654-5p, and miR-662 (**Figures 4A, B**). Next, a formula termed TAMIS consisted of these eight miRNAs weighted by their regression coefficients in a penalized Cox model. By virtue of this formula, the TAMIS score of each patient was calculated. All patients were assigned into high- and low-risk groups according to the optimal cutoff value determined by “survminer” R package. As illustrated in **Figures 4C, D**, the high-risk group presented significantly dismal OS relative to the low-risk group in both GSE53870 (HR = 2.260 [1.573-3.246], Log-rank *P <*0.0001) and TCGA-CHOL (HR = 3.348 [1.815-6.177], Log-rank *P* =0.012). After controlling for available clinical traits including age, gender, AJCC stage, and histological grade, multivariate Cox regression analysis revealed TAMIS remained statistically significant in two cohorts (both *P <*0.05), which suggested TAMIS was an independent prognostic factor for ICC (**Figures 4E, F**). Furthermore, ROC analysis measured the discrimination of TAMIS, with AUCs of 1, 2, 3, 4, and 5 years were 0.711, 0.718, 0.746, 0.845, and 1.000 in GSE53870 (**Figure 4G**), and 0.914, 0.739, 0.728, 0.746, and 0.767 in TCGA-CHOL (**Figure 4H**), respectively. The C-index [95% confidence interval] were 0.678 [0.626-0.730] and 0.750 [0.655-0.845] in two cohorts, respectively. Additionally, our TAMIS model displayed a good calibration, with the predicted probabilities of OS at 1-5 years accurately, describing the true risk observed in both GSE53870 and TCGA-CHOL datasets (**Figure S2A**). DCA was performed to evaluate the clinical net benefit with and without TAMIS for predicting prognosis. As showed in **Figure S2B**, TAMIS was feasible for making valuable and informed judgements of the prognosis, suggesting its clinical utility.

### Validation of TAMIS *via* qRT-PCR

**Figure 5:**
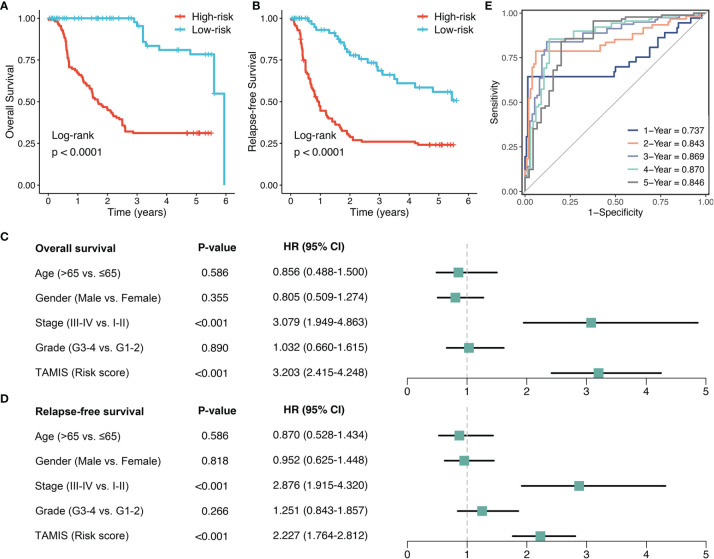
Validation of TAMIS *via* qRT-PCR. **(A, B)** Kaplan-Meier curves of OS **(A)** and RFS **(B)** according to TAMIS. **(C, D)**. Multivariable Cox regression analysis of OS **(C)** and RFS **(D)**. **(E)** Time-dependent ROC analysis for predicting OS at 1, 2, 3, 4, and 5 years.

To further validate the performance of TAMIS into a clinically translatable tool, we next quantified the expression of these eight miRNAs in a clinical in-house cohort of 181 CRC patients *via* qRT-PCR assay. **Figure S3A** illustrated the prognostic value of these eight miRNAs in our cohort, and all miRNAs were consistent with the previous results, with high expression predicting worse prognosis (all *P <*0.05). The Kaplan-Meier analysis also demonstrated that patients in the high-risk group exhibited pronouncedly unfavorable OS (HR = 3.174 [2.473-4.075], Log-rank *P <*0.0001, **Figure 5A**). In parallel, we evaluated the difference of relapse-free survival (RFS) between two groups, and observed the high-risk group also displayed a significantly worse RFS (HR = 2.357 [1.899-2.925], Log-rank *P <*0.0001, **Figure 5B**). Further multivariate Cox regression analysis revealed TAMIS remained the statistical significance for both OS and RFS, after adjusting for confounding features (**Figures 5C, D**). Thus, TAMIS can not only serve as an independent predictive biomarker of OS, but also have non-negligible predictive value for RFS. ROC analysis demonstrated a superior accuracy of TAMIS: the AUCs of 1, 2, 3, 4, and 5 years was 0.737, 0.843, 0.869, 0.870, and 0.846, respectively (**Figure 5E**). The C-index [95% CI] was 0.807 [0.749-0.866]. The calibration plot demonstrated the predicted probabilities of OS at 1-5 years accurately describing the true risk observed (**Figure S3B**). The DCA curve further validated its clinical utility (**Figure S3C**). Taken together, qRT-PCR assay verified the performance of TAMIS, which indicated that TAMIS had strong potential for clinical translation.

### Immune Landscape of TAMIS

**Figure 6:**
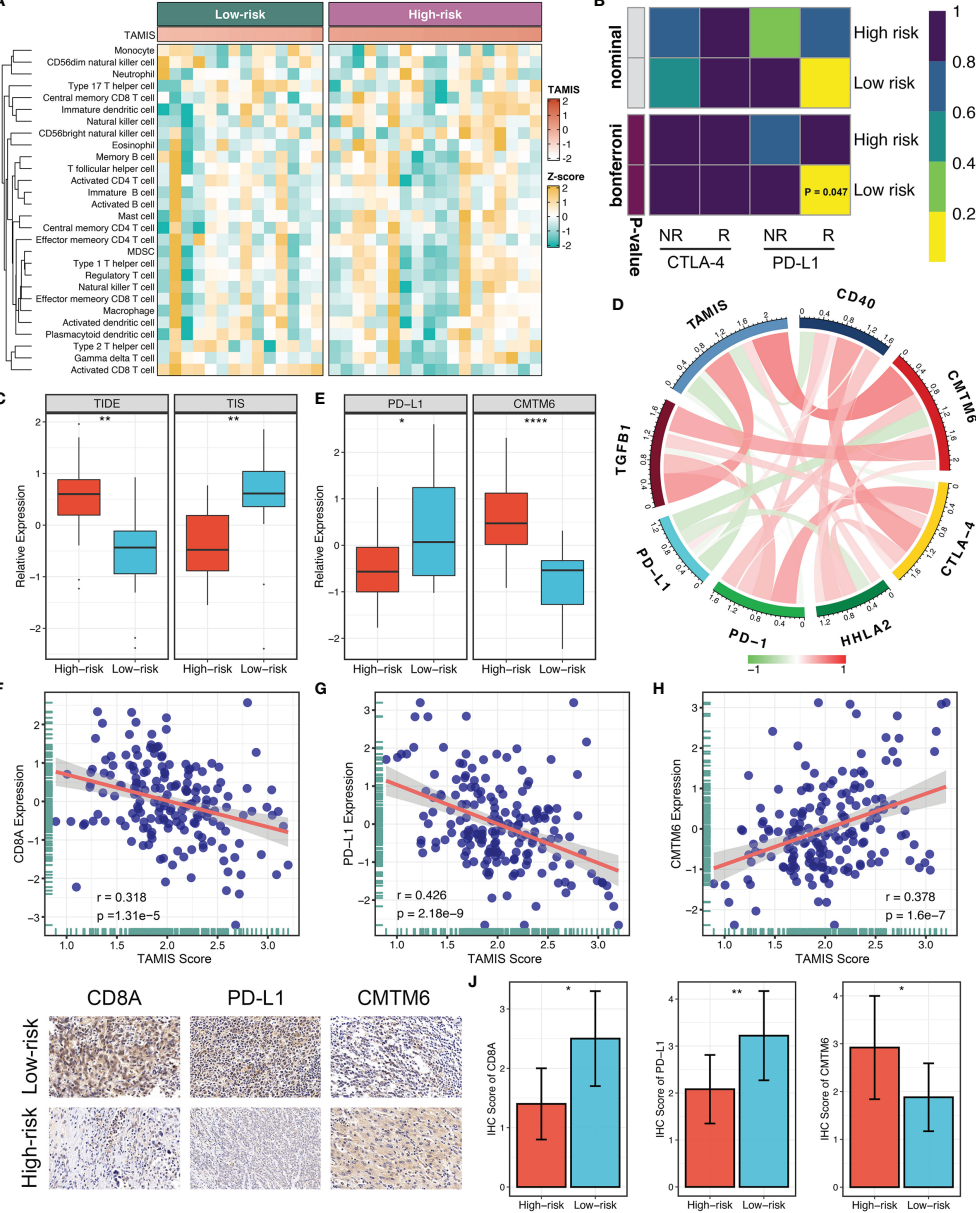
The immune landscape and immune checkpoint profiles of TAMIS. **(A)**. Relationships between TAMIS and immune cell infiltrations in TCGA-CHOL. **(B)** SubMap analysis manifested that the low-risk group could be more sensitive to the anti-PD-1 therapy (Bonferroni *P* =0.047). **(C)** Distribution difference of TIDE and TIS prediction score between the high-risk and low-risk groups. **(D)**. Correlations between TAMIS and PD-1, PD-L1, CTLA-4, CD40, HHLA2, TGFB1, and CMTM6 in TCGA-CHOL. **(E)** Distribution difference of PD-L1 and CMTM6 expression between the high-risk and low-risk groups. **(F–H)** Correlations between TAMIS and CD8A, PD-L1, and CMTM6 in our cohort. **(I)** Representative IHC staining images of CD8A, PD-L1, and CMTM6 between two risk groups. **(J)** Analysis of IHC scores between two risk groups according to the staining results of CD8A, PD-L1, and CMTM6. *P < 0.05, **P < 0.01, ****P < 0.0001.

Accumulating evidence revealed that TGF-β is a crucial enforcer of immune homeostasis and tolerance, exerting systemic immune suppression and inhibiting host immunosurveillance (8, 9). Therefore, we hypothesized that the two risk groups possessed distinct immune characterization. The heatmap illustrated the relative infiltration of 28 immune cells between two groups (**Figure 6A**). Patients in the low-risk group displayed a pronouncedly higher level of active CD8+ T cells, while patients in the high-risk group displayed a superior infiltration of natural killer T cells (**Figure S4A**). Of note, we also observed that the high-risk group presented a trend of high infiltration of many other cells but not significant, possibly due to the small sample size (**Figure S4A**). As is well-known, CD8+ T cells serve as the main and most important performer of killing tumor cells (29). Thus, the low-risk group with superior CD8+ cells might benefit more from ICI treatment. Furthermore, we applied three bioinformatics algorithms to evaluate the immunotherapeutic efficacy of two groups, including SubMap, TIDE, and TIS approaches. As shown in **Figure 6B**, SubMap revealed that the low-risk group displayed similar expression pattern with patients responding to PD-L1 inhibitors. Besides, patients in the low-risk group demonstrated the lower TIDE score and higher TIS score (both *P <*0.01), indicating that these patients were more likely to yield considerable clinical benefit from ICI treatment (**Figure 6C**).

### Immune Checkpoint Profiles of TAMIS

To gain insights into the underlying immune mechanism, we further explored the associations between our signature and seven promising immune checkpoints of ICC, including PD-1, PD-L1, CTLA-4, CD40, HHLA2, TGFB1, and CMTM6 (1, 30–33). As illustrated in **Figure 6D**, TAMIS possessed significant associations with these molecules, particularly PD-L1 and CMTM6. In TCGA-CHOL, patients in the high-risk group displayed the lower PD-L1 expression and higher CMTM6 expression compared with the low-risk group (**Figure 6E**). In this study, the superior level of CD8+ T cells infiltration and PD-L1 expression indicated that the low-risk group had a stronger potential to benefit from ICI treatment. Meanwhile, the higher expression CMTM6 might be a novel target for improving the prognosis of patients in the high-risk group. In addition, we also validated the relationships between TAMIS and CD8A, PD-L1, and CMTM6 in our cohort. Likewise, high TAMIS indicated lower expression levels of CD8A (*R* =0.318, *P <*0.0001) and PD-L1 (*R* =0.426, *P <*0.0001), and higher expression level of CMTM6 (*R* =0.378, *P <*0.0001) (**Figures 6F–H**). To further verify the protein expression in different levels of TAMIS, we performed IHC on paraffin sections which including 55 high-risk ICC and 52 low-risk ICC tissues. IHC images and scores demonstrated that the protein expression of CD8A and PD-L1 was dramatically higher in low-risk group, while CMTM6 significantly overexpressed in the high-risk group (**Figures 6I, J**).

### High TAMIS and Enhanced CMTM6 Expression Identified Super-Cold Tumors

**Figure 7:**
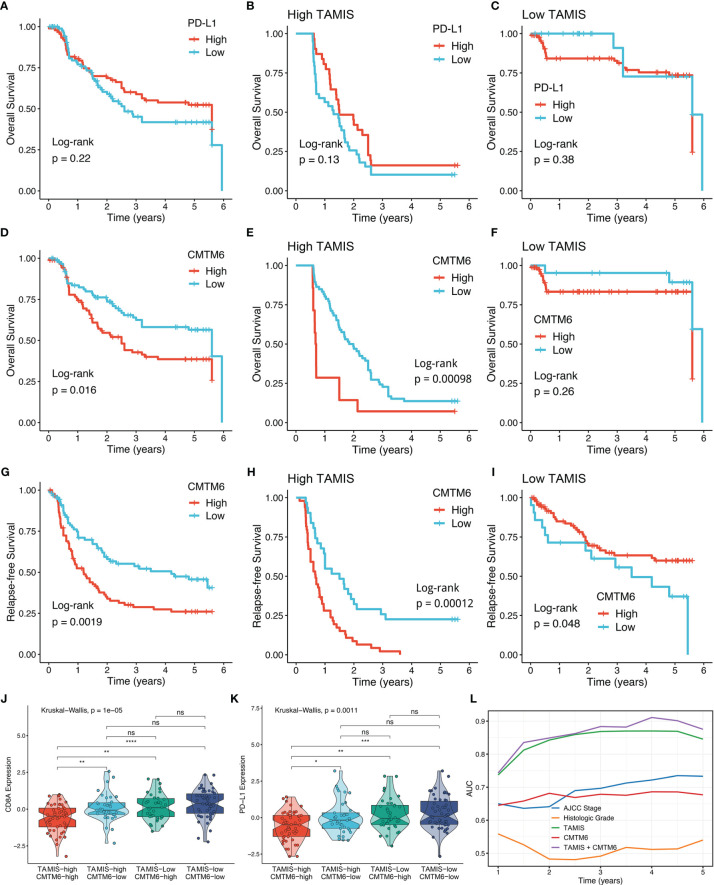
High TAMIS and enhanced CMTM6 expression identified super-cold tumors. **(A–C)** Kaplan-Meier curves of OS according to PD-L1 expression in all patients **(A)**, high TAMIS group **(B)**, and high TAMIS group **(C)**. **(D–F)**. Kaplan-Meier curves of OS according to CMTM6 expression in all patients **(D)**, high TAMIS group **(E)**, and low TAMIS group **(F)**. **(G–I)** Kaplan-Meier curves of RFS according to CMTM6 expression in all patients **(G)**, high TAMIS group **(H)**, and high TAMIS group **(I)**. **(J, K)** Distribution difference of CD8A **(J)** and PD-L1 **(K)** expression among four subtypes. **(L)** ROC curves of AJCC stage, histologic grade, TAMIS, CMTM6, and TAMIS+CMTM6 ns, P > 0.05 *P < 0.05, **P < 0.01, ***P < 0.001, ****P < 0.0001.

Consistent with previous study (31), PD-L1 expression failed to stratify OS and RFS in ICC (**Figure 7A** and **Figure S4B**). After stratification of TAMIS, there was still no significant difference regarding both OS and RFS between two distinct expression levels of PD-L1 (**Figures 7B, C** and **Figure S4C-D**). Conversely, high CMTM6 expression was dramatically related to poor OS and RFS without or with stratification of TAMIS (**Figures 7D–I**). This suggested that CMTM6, which positively correlated with TAMIS, could also further stratify TAMIS, with patients possessing low levels of TAMIS and CMTM6 having the best prognosis, while those possessing high levels of TAMIS and CMTM6 having the worst prognosis. Of note, the TAMIS^high^CMTM6^high^ subtype had pronouncedly lower expression levels of CD8A and PD-L1, whereas there was no significant difference between the other three subtypes (**Figures 7J, K**). This indicated that the TAMIS^high^CMTM6^high^ subtype might behave as “super-cold” ICC. The “super-cold” tumors have been defined by Chen et al. as a subtype with the lowest expression of T cell markers in different immune checkpoint subgroups (34). In ROC analysis, TAMIS achieved relative higher AUC in predicting the prognosis than AJCC stage, histologic grade, and CMTM6 alone (**Figure 7L**). Intriguingly, the improved discriminative ability was observed when CMTM6 was combined with TAMIS, although it was slightly elevated (**Figure 7L**).

## Discussion

Accumulating evidence indicates that miRNAs are closely implicated in modifying the TGF-β signaling (14–16). However, to date, only a few examples have been identified, and TGF-β-derived miRNAs and their potential value in improving clinical outcomes remain largely unexplored in ICC. In the present study, we introduced an integrated framework that enables the identification of TGF-β-derived miRNAs in ICC (termed “TGFmitor”). A total of 36 miRNAs were identified, which also could serve as a resource for deciphering the regulation of TGF-β signaling in ICC. We focused on their translational value in facilitating clinical management and found TGF-β-derived miRNAs demonstrated profound impacts on the prognosis of patients with ICC. In the present study, we were concerned that a single cohort with small size sample might identify many false-positive prognostic-related miRNAs, thus the screening criteria have been elevated as previous studies (35, 36). In total, nine miRNAs that pronouncedly associated with OS and aberrantly expressed in ICC were further determined for signature discovery.

As a popular machine learning algorithm, LASSO believes that sparseness is the truth of world, that is, useful information is quite limited, although the world is full of complex and diverse information (37). Thus, an ideal model should consist of a small number of variables but with strong extrapolation. Ultimately, our study selected eight miRNAs, including miR-135a-5p, miR-3195, miR-33b-3p, miR-380-3p, miR-380-5p, miR-548-3p, miR-654-5p, and miR-662, to fit a simple LASO model: TAMIS. All miRNAs within the TAMIS have been reported to be tightly associated with the progression and clinical outcomes across various cancer types (38–43). For example, miR-380-5p inhibits p53 to determine cell survival and is related to unfavorable clinical outcomes in MYCN-amplified neuroblastoma (40). miR-654-5p could advance the proliferation, metastasis, and chemoresistance of oral squamous cell carcinoma *via* targeting Ras/MAPK signaling (43).

In GSE53870 and TCGA-CHOL, TAMIS was proven to be an independent risk indicator for OS. To avoid false-positive results from sequencing data and test the clinical interpretation of TAMIS, another validation based on qRT-PCR results from 181 frozen ICC tissues further confirming the in-silico findings in clinical settings. In our data, TAMIS was also observed to independently predict the RFS of ICC. In addition, TAMIS displayed stable and accurate performance in three cohorts, with satisfactory AUCs (1-5 years) and C-index. Notably, this performance was superior to conventional AJCC stage and histological grade in predicting prognosis. Hence, our signature could be a promising surrogate for evaluating the prognosis of ICC in clinical settings.

Emerging evidence demonstrated that TGF-β is a crucial enforcer of immune homeostasis and tolerance, exerting systemic immune suppression and inhibiting host immunosurveillance (8, 9). Therefore, we further delineated the immune landscape of TAMIS, and found low TAMIS score indicated a superior level of CD8A expression by qRT-PCR and IHC assays. As is well-known, CD8+ T cells serve as the main and most important performer of killing tumor cells (29). The abundant infiltration of CD8+ T cell was the resource for ICI treatment. Additionally, we also observed that TAMIS was inversely associated with PD-L1 expression. Thus, patients with low TAMIS score possessed a stronger potential to benefit from ICI treatment. Three advanced algorithms, SubMap, TIDE, and TIS, further verified this assumption. On the other hand, CMTM6, a novel checkpoint molecule that regulates PD-L1 expression (44), was upregulated in patients with high TAMIS score. This finding suggested that CMTM6 might be a latent therapeutic target to improve the prognosis of patients in the high-risk group.

In line with previous study (31), PD-L1 expression failed to stratify OS and RFS in ICC. After stratification of TAMIS, there was still no significant difference in both OS and RFS between two distinct expression levels of PD-L1. Conversely, high CMTM6 expression was dramatically related to poor OS and RFS without or with stratification of TAMIS. This suggested that CMTM6 could further stratify TAMIS in ICC. The TAMIS^low^CMTM6^low^ subtype presented the best OS and RFS, while the TAMIS^high^CMTM6^high^ subtype had the worst OS and RFS. Notably, the TAMIS^high^CMTM6^high^ subtype also displayed significantly lower levels of CD8A and PD-L1 expression relative to the other subtypes. This indicated that the TAMIS^high^CMTM6^high^ subtype might behave as “super-cold” tumors, which are insensitive to immunotherapies due to the absence of CD8+ T cells and PD-L1. For these patients, the urgent task is to induce sufficient T cells, such as induction of immunogenic cell apoptosis, neoantigen vaccines, CTLA-4 inhibitors, CD40 agonists, and CAR-T, to further improve their clinical outcomes. More importantly, the improved discriminative ability was observed when CMTM6 was combined with TAMIS. These findings demonstrated that the combination of TAMIS and CMTM6 could better stratify ICC patients and facilitate the clinical management.

To the best of our knowledge, this is the first study to comprehensively identify TGF-β-derived miRNAs and explore their potential clinical significance in ICC. Prior to this study, a few reports constructed molecular signatures for predicting prognostic risk of cholangiocarcinoma (45–47). In comparison with these studies, our work focused on ICC rather than all cholangiocarcinoma, avoiding the bias of other cholangiocarcinoma types with significantly distinct biological characteristics; In parallel, qRT-PCR results for 181 patients were utilized to validate the performance and clinical interpretation of TAMIS. Notwithstanding TAMIS is promising in ICC, a few limitations should be acknowledged. First, despite all miRNAs within the TAMIS are implicated in the initiation and progression of various cancer types, their functions in ICC remain elucidated and further experiments are necessary. Second, all the samples from two datasets and our cohort were retrospective, and future validation of TAMIS should be performed in prospective multicenter study. Third, this study lacked eligible patients treated with ICIs, thus the implications of TAMIS for immunotherapy need to be further verified in ICC.

Taken together, our study introduced an integrated framework, “TGFmitor”, for systematically identifying the potential miRNA modulators of TGF-β signaling. Our study also established a feasible and reproducible TGF-β-derived miRNA signature, which possessed a stable and accurate performance in predicting the prognosis of ICC. With the immune landscape and immune checkpoint profiles of TAMIS, our signature could serve as a powerful tool to further optimize decision-making in prognosis and immunotherapeutic management.

## Data Availability Statement

Public data used in this work can be acquired from the TCGA Research Network portal (https://portal.gdc.cancer.gov/) and GSE53870 (http://www.ncbi.nlm.nih.gov/geo/). The raw experimental data supporting the conclusions of this article will be made available by the corresponding author.

## Ethics Statement

The studies involving human participants were reviewed and approved by The Ethics Committee Board of The First Affiliated Hospital of Zhengzhou University. The patients/participants provided their written informed consent to participate in this study.

## Author Contributions

ZL and XH designed this work. ZL integrated and analyzed the data. ZL wrote this manuscript. ZL, SW, XH, LW, LL, YZ, CG, QD, ZX, TL, and XH edited and revised the manuscript. All authors contributed to the article and approved the submitted version.

## Funding

This study was supported by the National Natural Science Foundation of China (81972663); Henan Province Young and Middle‐Aged Health Science and Technology Innovation Talent Project (YXKC2020037); and Henan Provincial Health Commission Joint Youth Project (SB201902014).

## Conflict of Interest

The authors declare that the research was conducted in the absence of any commercial or financial relationships that could be construed as a potential conflict of interest.

## Publisher’s Note

All claims expressed in this article are solely those of the authors and do not necessarily represent those of their affiliated organizations, or those of the publisher, the editors and the reviewers. Any product that may be evaluated in this article, or claim that may be made by its manufacturer, is not guaranteed or endorsed by the publisher.
