# Supplementary material for: Computational Recognition and Clinical Verification of TGF-β-Derived miRNA Signature With Potential Implications in Prognosis and Immunotherapy of Intrahepatic Cholangiocarcinoma
Source: Front Oncol. 2021 Oct 25;11:757919. doi: 10.3389/fonc.2021.757919 (PMC8573406; doi:10.3389/fonc.2021.757919)
Supplement: Supplementary file 1 [file DataSheet_1.docx]

**Supplementary material**

**Quantitative real-time PCR (qRT-PCR)**

Total RNA was isolated from ICC tissues and paired adjacent non-cancerous tissues with RNAiso Plus reagent (Takara, Dalian, China), according to the manufacturer's instructions. The RNA quality was evaluated using a NanoDrop One C (Waltham, MA, USA), and the RNA integrity was assessed using agarose gel electrophoresis. An aliquot of 1 µg of total RNA was reverse-transcribed into complementary DNA (cDNA) using a High-capacity cDNA Reverse Transcription kit (TaKaRa Bio, Japan), according to the manufacturer's protocol. miRNAs were reverse transcribed using a miRNA reverse transcription kit (TaKaRa Bio, Japan). This project was approved by the Ethics Committee Board of The First Affiliated Hospital of Zhengzhou University.

In the qRT-PCR analysis, the enrolled eight miRNAs in the TAMIS signature and feature genes (including CD8A, PD-L1, and CMTM6) were detected. qRT-PCR was performed using SYBR Assay I Low ROX (Eurogentec, USA) and SYBR® Green PCR Master Mix (Yeason, Shanghai, China) to detect gene expression. The 2^-ΔΔCt^ method was used to calculate the relative levels of gene and miRNA expression, and then log2 transformed for subsequent analysis. The primers are listed in Table S2. GAPDH or U6 was used as the endogenous control for normalization. qRT-PCR assays were performed in triplicate with the following conditions: (1) 95 °C for 5 min and (2) 40 cycles of 95 °C for 10 s and 60 °C for 30 s. The relative expression of LINC01272 was calculated using the ΔCT (Ct lncRNA-Ct GAPDH) method. miRNA qPCR was carried out according to a miRNA qPCR kit (TaKaRa Bio, Japan), and U6 was used as the internal reference.
