# Supplementary material for: Computational Recognition and Clinical Verification of TGF-β-Derived miRNA Signature With Potential Implications in Prognosis and Immunotherapy of Intrahepatic Cholangiocarcinoma
Source: Front Oncol. 2021 Oct 25;11:757919. doi: 10.3389/fonc.2021.757919 (PMC8573406; doi:10.3389/fonc.2021.757919)
Supplement: Supplementary file 2 [file DataSheet_2.docx]

**Supplementary Figure**

- **Figure S1**
- **Figure S2**
- **Figure S3**
- **Figure S4**

**
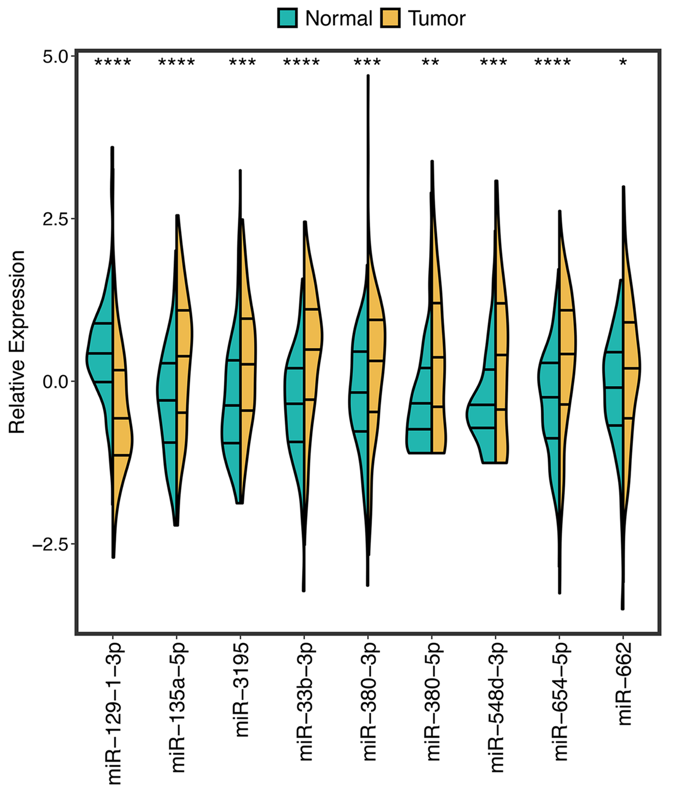
**

**Figure S1.** Distribution of nine miRNAs expression between normal and tumor tissues via qRT-PCR assays. **P* <0.05, ***P* <0.01, ****P* <0.001, *****P* <0.0001.


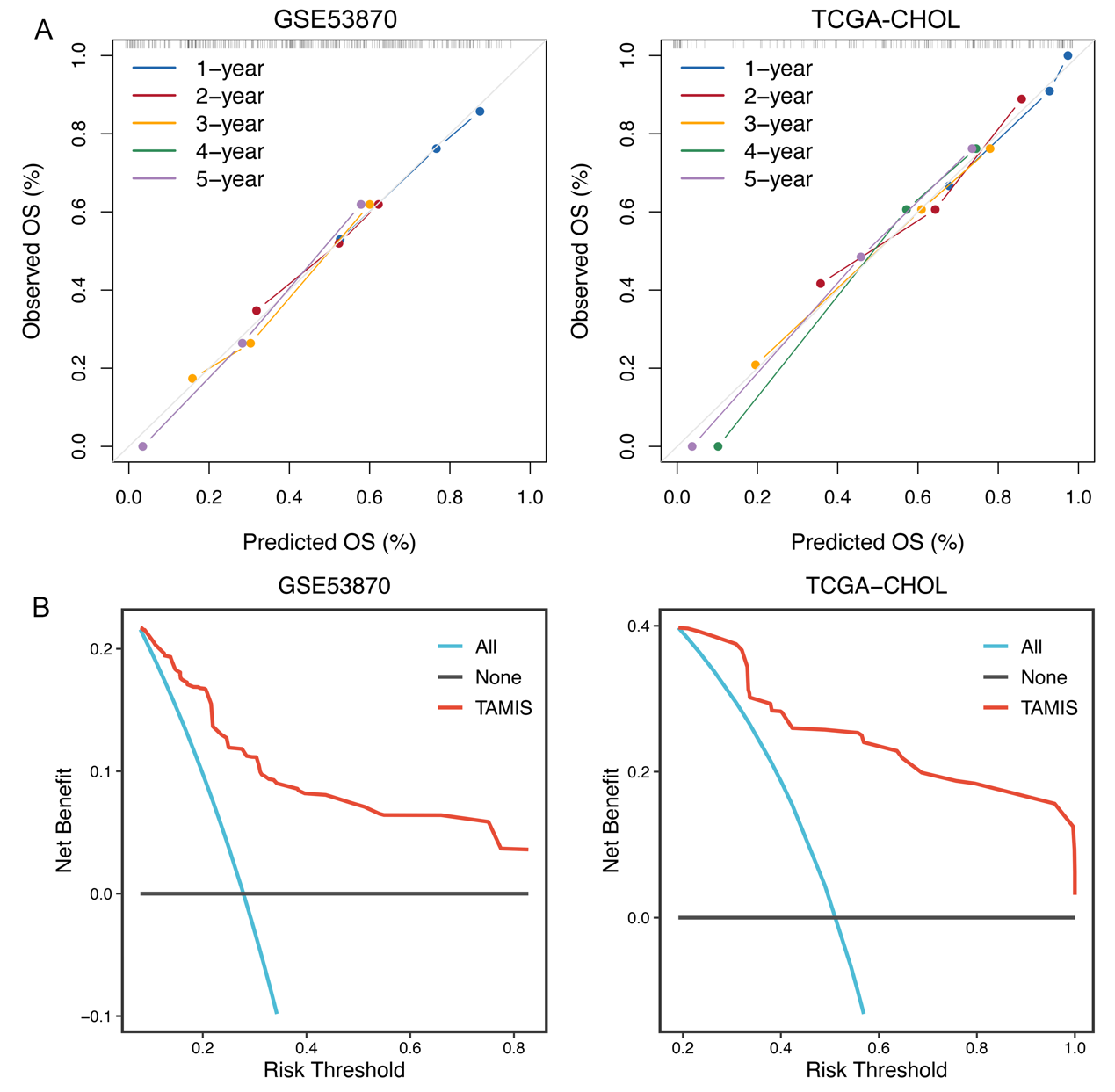


**Figure S2.** The calibration and DCA curves in GSE53870 and TCGA-CHOL. **A**. Calibration plots in two cohorts. **B**. DCA curves in two cohorts.


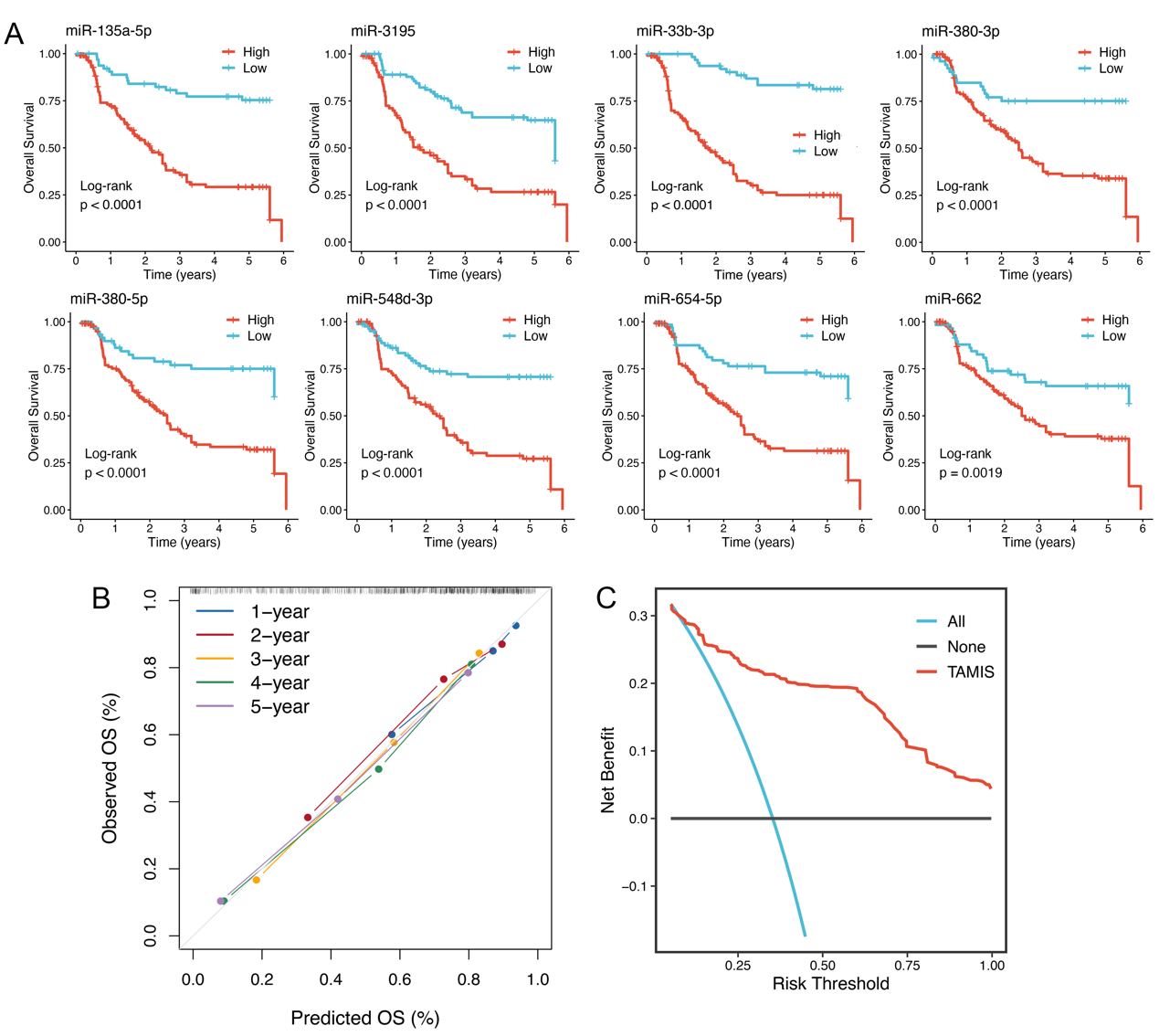


**Figure S3.** Assessment of eight miRNAs and TAMIS in our cohort. **A**. Kaplan-Meier analysis of eight miRNAs in TAMIS. **B**. Calibration plot. **C**. DCA curve.


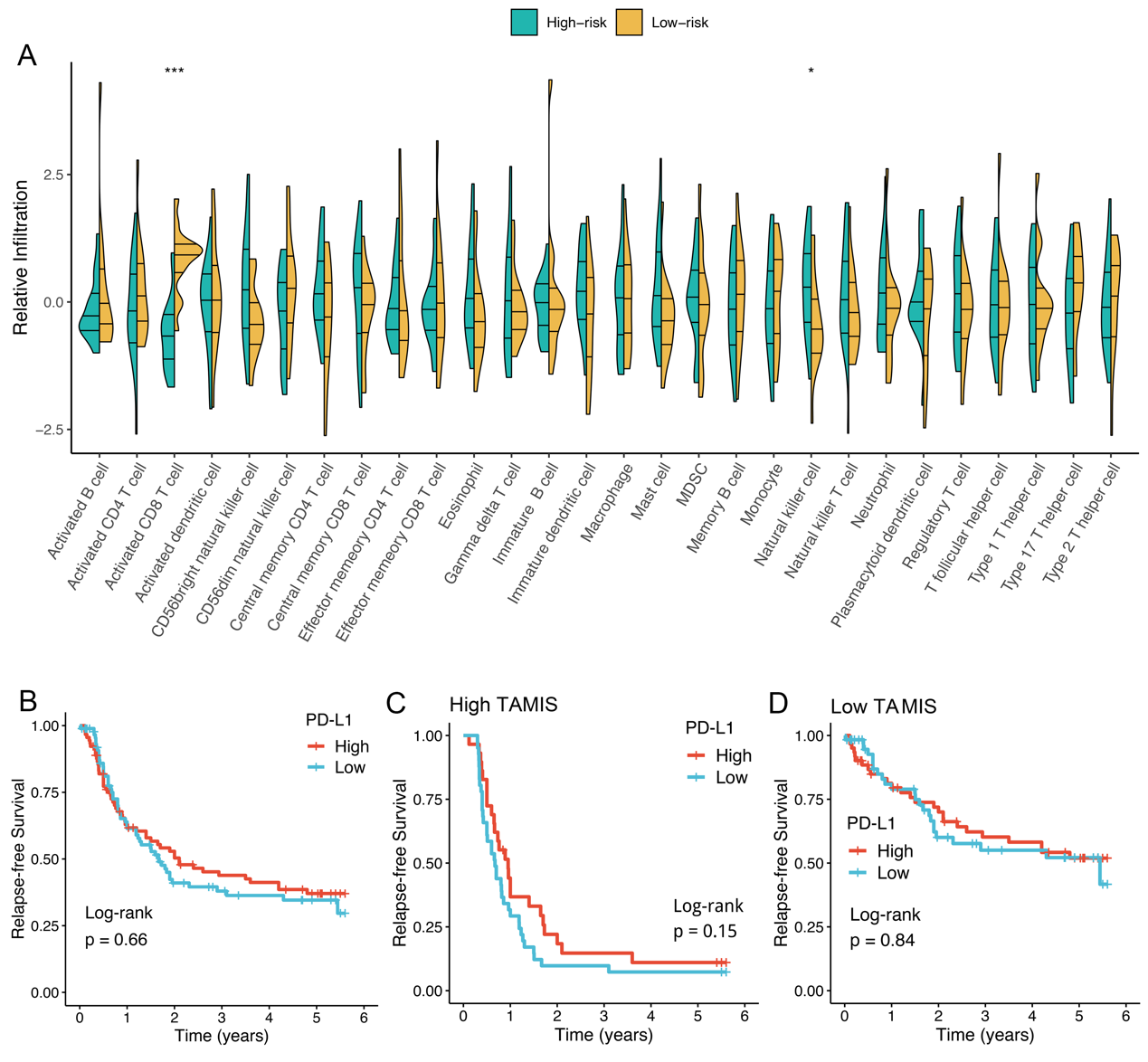


**Figure S4.** Distribution of 28 immune cell infiltrations and Kaplan-Meier curves of RFS according to PD-L1 expression**. A**. Distribution of 28 immune cell infiltrations between two risk groups. ****P* <0.001. **B-D**. Kaplan-Meier curves of RFS according to PD-L1 expression in all patients (**B**), high TAMIS group (**C**), and low TAMIS group (**D**).
